# A Case of Supplement-Induced Hepatitis

**DOI:** 10.7759/cureus.30433

**Published:** 2022-10-18

**Authors:** Carolina M Pusec, Ryan Wolsky, Christopher Llerena, Parimal Sura

**Affiliations:** 1 Internal Medicine, Advocate Christ Medical Center, Oak Lawn, USA; 2 Internal Medicine, University of Illinois at Chicago, Chicago, USA

**Keywords:** acute hepatitis, herbal supplements, ashwagandha, steatohepatitis, hepatitis

## Abstract

Acute hepatitis is an uncommon sequela of herbal supplement use. Regardless, considering the hepatotoxic effects of natural supplements is important, especially in patients taking other medications or substances. We herein describe a case of acute steatohepatitis in a patient who chronically consumed high doses of ashwagandha and other herbal supplements in the context of alcohol use and a multi-medication regimen.

## Introduction

The use of natural medicines and herbal supplements has grown in popularity worldwide given their greater affordability over prescription medications, less perceived adverse effects compared to synthesized medicines, and their advertised beneficial effects against various health ailments [1]. However, what is often overlooked are the potential toxic effects, especially when used in combination with other herbal supplements, prescription drugs, over-the-counter medications, or the use of alcohol or illicit drugs [2-5]. Recently, cases of herbal supplement-induced hepatitis, particularly with ashwagandha, have been reported [6-8]. Here, we report a case of acute hepatitis in a 43-year-old woman who consumed an assortment of supplements, including ashwagandha, in combination with mild alcohol and ibuprofen consumption after an acute ankle injury.

## Case presentation

A 43-year-old female with a past medical history of iron deficiency anemia presented with diffuse abdominal pain and jaundice for the past three days. She described the pain as a constant, dull, heavy pressure localized to the epigastrium. She also reported three weeks of severe fatigue and dark urine. She denied any viral symptoms, dysuria, pruritus, changes in stool frequency or consistency, nausea, fevers, or chills. She was originally from Ecuador but moved to the United States when she was one year old and has had no recent travel. She drinks two glasses of wine two to three times per week. She sprained her ankle a week and a half prior to hospitalization and had been taking 1200 mg of ibuprofen daily for the pain since then. She has taken daily supplements of maximum strength (2100 mg) ashwagandha, dandelion tea, vitamin E, and blood builders, which include iron, vitamin B12, and folate, for more than a year.

On admission, her BMI was 22.71 and a physical exam showed scleral icterus, grossly apparent jaundiced skin, and +2 bilateral leg edema. Initial workup showed elevated levels of aspartate transaminase (AST), alanine transaminase (ALT), alkaline phosphatase, and markedly elevated total bilirubin and direct bilirubin with low albumin (see Table 1). In addition, she had a negative viral hepatitis panel and testing for autoimmune causes including anti-mitochondrial, anti-smooth muscle, and antinuclear antibodies (ANA) were all negative. Right upper quadrant ultrasound demonstrated increased liver echogenicity suggestive of hepatic steatosis. There was no evidence of mass lesions, abnormalities associated with intrahepatic biliary ducts, an impedance of flow through the portal vein, or evidence of choledocholithiasis. Further radiography with computed tomography of the abdomen and pelvis (CTAP) scan further confirmed these findings and additionally showed prominent porta hepatis lymph nodes, which suggest a likely reactive process. Magnetic resonance cholangiopancreatography (MRCP) without contrast revealed a liver span of 26.1 cm and no evidence of filling defects or strictures of the biliary tree.

**Table 1:** Patient’s liver function tests prior to her hospital stay (baseline) and on day one and day four.

Lab values	Baseline (6/26/19)	Day 1 (11/01/21)	Day 4 (11/04/21)
Alanine transaminase (IU/L)	48	70	49
Aspartate transaminase (IU/L)	123	195	166
Alkaline phosphatase (IU/L)	72	422	316
Total bilirubin (mg/dL)	0.5	13.8	14.3
Direct bilirubin (mg/dL)	-	11.5	-
Albumin (g/dL)	3.3	1.9	1.7
International normalized ratio	1.3	-	-

**Figure 1:**
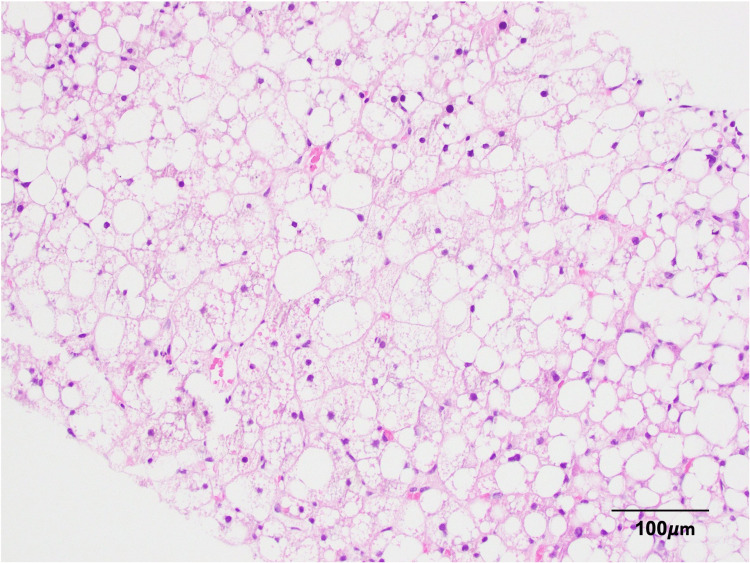
Liver biopsy with hematoxylin and eosin stain at 200x magnification.

Given the lab values were highly suggestive of cholestatic disease with largely negative radiography findings, a liver biopsy was pursued. The histologic findings were consistent with steatohepatitis with predominantly sinusoidal and periportal fibrosis (stage 2). There was marked macrovesicular and microvesicular steatosis present throughout >90% of the liver parenchyma and megamitochondria. There was no evidence of ballooned hepatocytes or Mallory-Denk bodies (see Figure 1).

## Discussion

Given the growing popularity of herbal supplements, it is necessary to understand the limitations and potential adverse effects of these compounds individually or in combination with other medications, supplements, and alcohol. Aloe vera-induced hepatotoxicity has been reported widely in countries such as Korea, Germany, and the United States, among many others [6-8]. Other supplements, such as ashwagandha, have also been shown to cause hepatitis, particularly when taken in conjunction with additional supplements or drugs [9-11]. While it is difficult to discern whether ashwagandha alone is sufficient to cause hepatitis, more cases are emerging in patients who are consuming more than one type of supplement or taking supplements with concomitant use of alcohol or other prescribed or non-prescribed medications. Many of the current studies support the beneficial effects of ashwagandha for treating anxiety and depression or boosting testosterone. However, conclusions are limited and reports on the adverse effects are scarce given their small sample sizes and lack of long-term use and follow-up [12-15]. In this case report, the patient had negative diagnostic labs and imaging studies for viral, autoimmune, neoplastic, or structurally abnormal biliary system etiologies. However, the patient displayed signs of cholestatic liver disease evidenced by her elevated liver function tests, alkaline phosphatase, direct bilirubin, and liver biopsy revealing varying vesicular steatosis with sinusoidal and periportal fibrosis. We speculate that acute ingestion of ibuprofen due to her ankle injury exacerbated an already existing low-grade hepatitis caused by long-term use of supplements and alcohol consumption enough to result in signs and symptoms of acute cholestatic steatohepatitis.

## Conclusions

The use of herbal supplements has been growing and continues to be significantly utilized in lieu of or as an adjunct to prescription medications globally. More reporting of liver function and damage after the use of herbal supplements is needed to better assess potential hepatic adverse effects. Variables such as the dosages, duration of consumption, and manufacturers of a given herbal supplement are all important points to also consider and the prescribing physician should be aware of the hepatotoxic effects of herbal supplements, especially in the context of poly-supplement/substance use, which is highlighted in this case report.
